# Relationships Between Disease Severity and the C-reactive Protein/Albumin Ratio and Various Hematological Parameters in Patients With Acne Vulgaris

**DOI:** 10.7759/cureus.44089

**Published:** 2023-08-25

**Authors:** Erdal Pala, Mustafa Bayraktar

**Affiliations:** 1 Department of Dermatology and Venerology, Faculty of Medicine, Ataturk University, Erzurum, TUR; 2 Department of Family Medicine, Faculty of Medicine, Ataturk University, Erzurum, TUR

**Keywords:** c-reactive protein (crp), inflammation, hematological parameters, severity of disease, crp/albumin ratio, acne vulgaris knowledge, albumin level

## Abstract

Objective: Acne vulgaris, an inflammatory disease, was investigated in this study with the claim that the C-reactive protein (CRP)/albumin ratio and some hematological parameter ratios have the potential to be used as inflammatory markers to monitor disease severity and prognosis.

Methods: A descriptive cross-sectional study was conducted with two groups of patients aged 18-65 years, 61 patients diagnosed with acne vulgaris and 35 healthy control patients, and routine hemogram and biochemical parameters were compared. The Global Acne Severity Index was used to determine the severity of acne vulgaris.

Results: The median age of acne patients was 22.0 (IQR=5.0) and the median age of healthy volunteers was 30.0 (IQR=14.0). There were 75.4% (n=46) women in the acne group and 77.1% (n=27) women in the control group. Among the acne patients, 42.6% (n=26) had mild acne, and 41% (n=25) had moderate acne severity. A significant difference was found between the study groups in terms of CRP/albumin ratio, CRP, monocyte/lymphocyte ratio (MLR), lymphocyte/albumin ratio (LAR), and monocyte/CRP ratio (MCR) according to laboratory test results and calculated test ratios (p<0.05). The area under the curve (AUC) value of the CRP/albumin ratio in the receiver operating characteristic (ROC) analysis between the acne and control groups was 0.660, and its cut-off value was found to be 0.236 with 68.6% sensitivity and 68.9% specificity.

Conclusion: This study is the first to compare the CRP/albumin ratio with the severity of acne vulgaris in the literature. CRP/albumin ratio and MCR may serve as inflammatory markers that can be used to monitor the severity of acne vulgaris.

## Introduction

Acne vulgaris (AV) is a chronic inflammatory condition that primarily affects the pilosebaceous unit, consisting of the hair follicle and sebaceous glands. Increased sebum production in the pilosebaceous unit, colonization by Cutibacterium acnes, and subsequent inflammation all play crucial roles in the pathogenesis. Inflammation leads to the formation of typical acne lesions, depending on its severity. The more severe and prolonged the inflammation, the higher the risk of fibrous tissue proliferation and scar formation. Clinically, AV manifests as various lesions primarily located on the back, chest, and face [1]. The condition is common in the young population and can significantly impair patients' quality of life [2]. Recent studies have shown that various hematological parameters, which rise with inflammation, can be used as markers to monitor the severity of AV. These include the neutrophil/lymphocyte ratio (NLR), C-reactive protein (CRP), and mean platelet volume (MPV) [3]. However, no studies have investigated the relationship between the CRP/albumin ratio (CAR) and disease severity in AV. The aim of this study was therefore to investigate the usefulness of CAR and other hematological parameter ratios as potential inflammatory markers in determining the severity of AV and thus to contribute to the existing literature.

## Materials and methods

Ethical approval

The study was conducted in accordance with the Helsinki Declaration, good clinical practice, once local ethics committee approval had been granted (no. 567, dated December 30, 2021).

Clinical trial registry

This study was registered with the ID number NCT06004583.

Study design

The research was conducted as a descriptive, single-center, cross-sectional study. For the study, acne patients were selected from the patients who applied to our dermatology and venereal diseases outpatient clinic between January and May 2022 and were clinically diagnosed by a dermatologist. Healthy control group participants were recruited from a Family Health Center. At the beginning of the study, a 1:1 allocation was made for both acne patients and healthy control groups, and it was determined that 61 patients from the acne group and 56 people from the healthy group were found to be eligible and participated voluntarily. However, in order to have similar demographic variability, age, and gender distribution in both groups, some patients, especially those over the age of 40, were excluded from the study, and a total of 21 people in the healthy control group were not included in the study. As a result, 61 acne patients and 35 healthy people were included in the study.

The patients’ demographic characteristics, duration of symptoms, and smoking status were recorded. A physical examination was performed, and a description of existing lesions was documented. Routine complete blood count and biochemical parameters were requested. For AV patients, patients under the age of 18, pregnant and breastfeeding patients, those using systemic antibiotics or anti-inflammatory drugs within 15 days, those with acute and chronic infections, those with a history of rheumatologic, malignancy, autoimmune disease, and other inflammatory skin diseases were excluded from the study. The Global Acne Severity Index was used to determine the severity of AV, with scores ranging from 1 to 18 categorized as mild, 19 to 30 as moderate, 31 to 38 as severe, and scores of 39 or higher as very severe. Leukocyte, neutrophil, lymphocyte, platelet, and monocyte parameter values obtained from routine complete blood count tests, as well as the NLR, monocyte/lymphocyte ratio (MLR), lymphocyte/albumin ratio (LAR), monocyte/CRP ratio (MCR), neutrophil/monocyte ratio (NMR), and platelet/lymphocyte ratio (PLR) calculated using those values, were included in the analysis. CAR and CRP-albumin ratios were calculated and recorded for both groups. Our hospital laboratory reference values were taken into account in the evaluation of laboratory tests. Normal values were defined as follows: CRP <5 mg/L, albumin 3.5-5.2 g/dL, WBC 3.9-10.8 (10^3^/μL), lymphocyte 1.16-3.61 (10^3^/μL), neutrophil 1.91-7.61 (10^3^/μL), monocyte 0.28-0.96 (10^3^/μL), and platelet 145-345 (10^3^/μL). Five cubic centimeter blood samples were drawn from all patients for the determination of CRP and albumin levels. These were then centrifuged at 3,500 rpm for 15 minutes and stored at -80°C until the day of analysis. CRP, albumin, and CAR were measured using a Cobas 702 biochemistry autoanalyzer (Roche® Diagnostics, Mannheim, Germany) on the study day.

Statistical analysis

The study data was analyzed on SPSS version 23.0 (IBM, Armonk, NY, USA) software. The Kolmogorov-Smirnov test was used to evaluate the normality of data distribution. Categorical data were presented as frequencies and percentages and numeric data as mean and standard deviation or median and interquartile ranges (IQR), depending on the normality of distribution. Student's t-test or the Mann-Whitney U test was applied for comparisons between two groups, depending on the normality of distribution. One-way ANOVA or the Kruskal-Wallis test was used for the analysis of three or more groups, again depending on the normality of distribution. Fisher's exact test was applied in the analysis of categorical data. Receiver operating characteristic (ROC) analysis was applied to determine the area under the curve (AUC) and cut-off values for CAR. A p-value <0.05 was considered statistically significant.

## Results

Sixty-one acne patients and 35 healthy controls were included in the study. Forty-six (75.4%) patients in the acne group and 27 (77.1%) members of the control group were female, although the difference was not statistically significant (p>0.05). Median age was significantly lower in the acne group, at 22.0 (IQR=5.0) years, than in the control group, at 30.0 (IQR=14.0) years (Table 1).

**Table 1:** Demographic properties and comparisons of the study groups * Pearson chi-square test † Mann-Whitney U test

Demographic properties		Acne Group	Control Group	P-value
Sex (Frequency, percent)	Female	46 (75.4%)	27 (77.1%)	0.828*
	Male	15 (24.6%)	8 (22.9%)	
	Total	61 (100%)	35 (100%)	
Age (Median, Interquartile Range (IQR))		22.0 (IQR=5.0)	30.0 (IQR=14.0)	<0.001^†^

**Figure 1:**
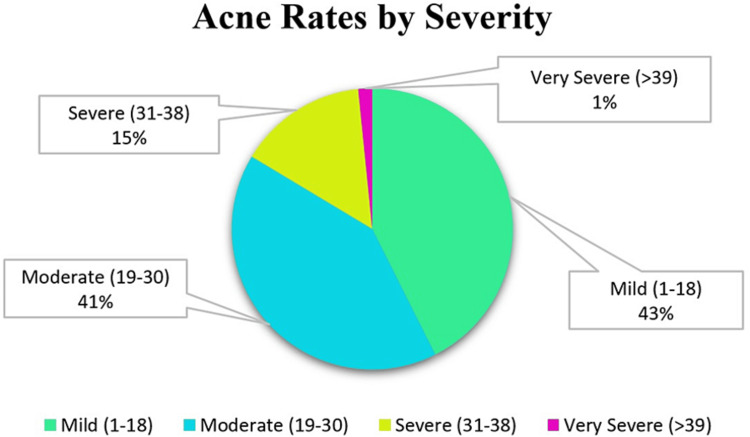
Acne rates according to acne severity in patients

Analysis revealed that 13.1% (n=8) of the acne group were smokers, with a median smoking duration of 6.30 ± 17.47 months. The median duration of acne symptoms was 24.0 (IQR=18.0) months. In terms of severity, 42.6% (n=26) of the patients had mild acne, and 41% (n=25) had moderate acne (Figure 1).

Comparisons of laboratory test results and calculated test ratios between the study groups are presented in Table 2. Significant differences emerged between the two groups’ CRP, CAR, MLR, LAR, and MCR values (p<0.05). CRP was significantly higher in the control group than in the acne group (p=0.014). However, when CRP was categorized as normal if below 5 mg/L and high if greater than 5 mg/L and a chi-square test was subsequently applied, no significant difference was observed between the two (p=0.097). Albumin levels differed insignificantly between the two groups (p=0.050), but all participants had albumin values within normal ranges (3.5-5.5 g/dL).

**Table 2:** Laboratory test analysis results and comparisons of acne and control group * Mann-Whitney U test † Student-t test ‡ Fisher’s Exact test WBC: white blood cell; CRP: C-reactive protein; CAR: CRP/albumin ratio; NLR: neutrophil/lymphocyte ratio; NMR: neutrophil/monocyte ratio; PLR: platelet/lymphocyte ratio; MLR: monocyte/lymphocyte ratio; LAR: lymphocyte/albumin ratio; MCR: monocyte/CRP ratio

Laboratory test parameters	Mean ± SD and Median, Interquartile Range (IQR)	Acne Group	Control Group	P-value
WBC	Median	7.23	7.77	0.343*
	Interquartile Range	2.16	2.83	
Platelet	Mean	281.23	295.40	0.247†
	Std. Deviation	70.66	48.11	
Neutrophil	Median	3.96	4.39	0.359*
	Interquartile Range	1.48	2.06	
Monocyte	Mean	0.57	0.57	0.932†
	Std. Deviation	0.13	0.19	
Lymphocyte	Mean	2.40	2.62	0.107†
	Std. Deviation	0.62	0.69	
Albumin	Median	4.72	4.60	0.050*
	Interquartile Range	0.41	0.47	
CRP	Median	0.73	1.22	0.014*
	Interquartile Range	1.34	0.46	
CRP category	Normal CRP	56 (91.8%)	35 (100%)	0.097‡
	Elevated CRP	5 (8.2%)	0 (0%)	
CAR	Median	0.15	0.28	0.009*
	Interquartile Range	0.30	0.10	
NLR	Median	1.77	1.65	0.586*
	Interquartile Range	0.86	0.91	
NMR	Median	7.56	8.03	0.246*
	Interquartile Range	2.46	3.70	
PLR	Mean	123.06	118.64	0.559†
	Std. Deviation	38.34	30.13	
MLR	Median	0.24	0.21	0.044*
	Interquartile Range	0.07	0.09	
LAR	Mean	0.51	0.58	0.045†
	Std. Deviation	0.14	0.17	
MCR	Median	0.85	0.42	0.009*
	Interquartile Range	1.01	0.26	

Comparisons of laboratory values based on the severity of acne according to the Acne Severity Index and the categorized groups are presented in Table 3. Only CRP, CAR, and MCR values exhibited significant differences between the groups based on disease severity, while platelet and albumin values differed significantly based on severity categories (p<0.05).

**Table 3:** Comparison of laboratory test results according to acne disease severity values and categories * One-way ANOVA test † Kruskal-Wallis test WBC: white blood cell; CRP: C-reactive protein; CAR: CRP/albumin ratio; NLR: neutrophil/lymphocyte ratio; NMR: neutrophil/monocyte ratio; PLR: platelet/lymphocyte ratio; MLR: monocyte/lymphocyte ratio; LAR: lymphocyte/albumin ratio; MCR: monocyte/CRP ratio

Laboratory test parameters	Acne disease severity levels	Acne disease severity categories
WBC	0.726*	0.346†
Platelet	0.022*	0.557*
Neutrophil	0.681*	0.385†
Monocyte	0.264*	0.940*
Lymphocyte	0.962*	0.368*
Albumin	0.032*	0.164†
CRP	0.859*	0.045†
CAR	0.885*	0.029†
NLR	0.936*	0.844†
NMR	0.147*	0.311†
PLR	0.193*	0.880*
MLR	0.812*	0.312†
LAR	0.930*	0.190*
MCR	0.984*	0.022†

**Figure 2:**
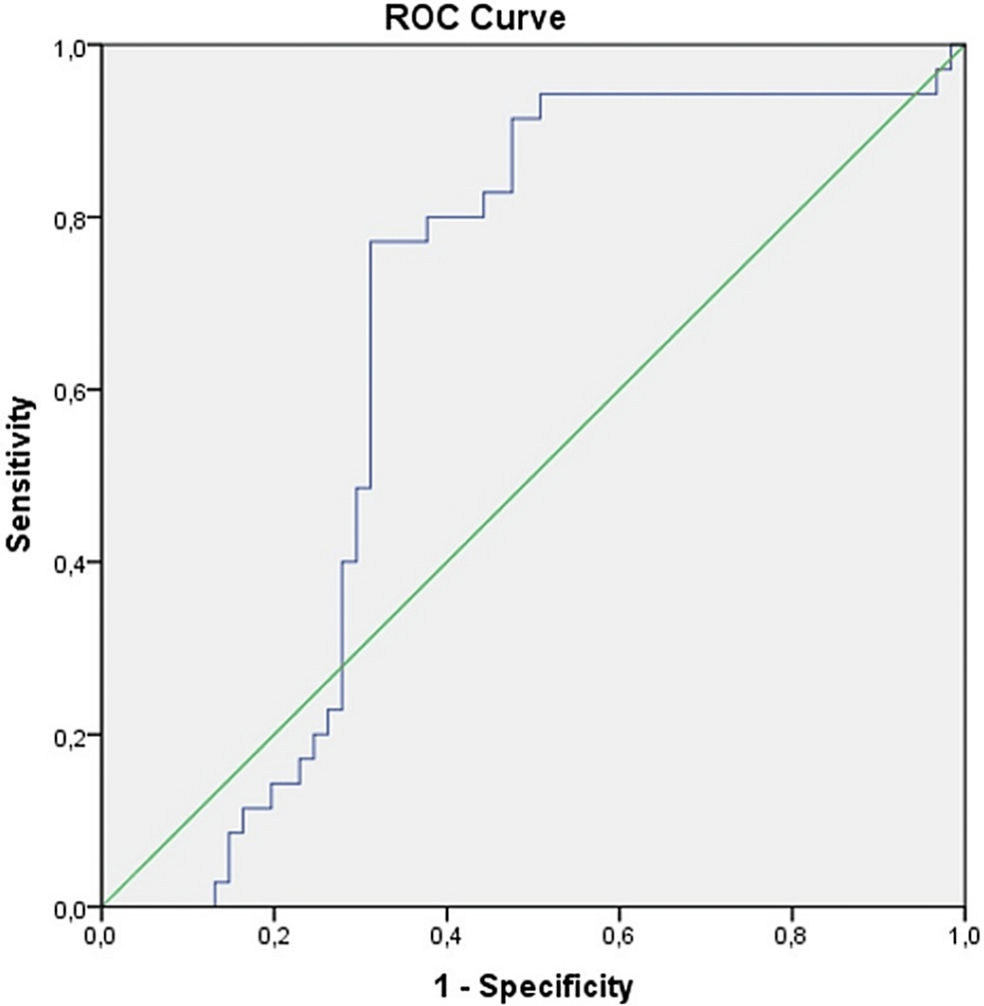
Curve of ROC analysis of CAR ROC - receiver operating characteristic, CAR - CRP/albumin ratio

ROC analysis of CAR values between the acne and control groups yielded an AUC of 0.660 (Table 4, Figure 2). The cut-off value for the CAR ratio was 0.236, with a sensitivity of 68.6% and a specificity of 68.9%.

**Table 4:** Area under curve (AUC) analysis of CAR ratio ^a^Under the nonparametric assumption ^b^Null hypothesis: true area = 0.5 CAR - CRP/albumin ratio

Area	Std. Error^a^	Asymptotic Sig.^b^	Asymptotic 95% confidence interval	
			Lower Bound	Upper Bound
0.660	0.058	0.009	0.547	0.773

## Discussion

AV is a chronic, disease with a complex pathogenesis in which inflammation plays a significant role. Damage to the pilosebaceous unit occurs in the etiopathogenesis due to colonization of Cutibacterium acnes, leading to the release of proteases, lipases, and chemokines. Following this damage, lymphocytes, monocytes, and neutrophils migrate to the affected area. Activation of toll-like receptor-2 on monocytes leads to the induction of interleukin-8 (IL-8) secretion [4]. Assessing the severity of inflammation in AV is crucial in predicting the development of morbidities. Intense inflammation entails an increased risk of fibrous tissue and scar formation. Easily applicable, simple, and inexpensive inflammatory markers are therefore needed to monitor the degree of inflammation in this patient group. The purpose of this study was to examine the relationship between CAR, MPV, LAR, and other hematological parameter ratios and the severity of AV, a subject that has not been reported in the literature to date.

CAR, obtained by dividing the positive acute-phase protein CRP by the negative acute-phase protein albumin, has emerged as an inflammatory marker in recent years. It is used to predict inflammation and is also of prognostic value [5,6]. CAR values differed significantly between the AV and healthy control groups in the present study. When focusing on detail, all CRP results of the participants in the control-healthy group had normal CRP levels and the median value was within the normal range. However, when we look at the CRP values of the acne group, some patients had higher CRP levels, but most of the acne patients (51 out of 61 acne patients) had mild or moderate acne severity index, which may not cause an increase in CRP level. Therefore, the overall mean value was in the normal reference ranges in the acne group, but it was found to be lower than the control group. Additionally, both groups had normal albumin levels, and therefore CAR level was only affected by CRP levels, and small differences in the CRP values affected the CAR value.

Considering the age difference of both groups, we preferred to include participants in the same age range, and the minimum and maximum ages of the study groups were similar, between 18-39 in the control group and between 18-40 in the acne group. However, when statistically analyzed, there was a difference between the groups. Since the elderly were not included in the study, it cannot be considered that this statistical difference caused a difference in CRP value between the groups. When we classify CRP levels as normal and high, the lack of difference between the groups as seen in Table 2 supports this view.

Examination of the relationship between disease severity and CAR revealed a significant difference among AV patients categorized based on disease severity and the control group. At ROC analysis, the AUC value for CAR was 0.660. The cut-off value for CAR was 0.236, with a sensitivity of 68.6% and a specificity of 68.9%. Based on these results, we anticipate that CAR may be capable of use as a diagnostic marker in AV. Although this study is the first to investigate CAR in patients with AV, a number of inflammatory markers have previously been reported in the context of the disease. One study investigated CRP, ferritin, and hepcidin levels in patients with AV and reported elevated levels of CRP and hepcidin in particular. Elevated CRP levels were correlated with disease severity [7]. Conversely, no relationship was found between CRP and AV severity in another study [8]. In the present research, when CRP values were categorized as normal if below 5 mg/L and high if above 5 mg/L, no significant difference was found between the AV and control groups. To the best of our knowledge, another parameter examined, MLR, has not appeared in the relevant literature to date. However, it has been investigated in patients with rosacea, with no significant difference being observed between a control group and a rosacea group [9]. A similar study involving patients with alopecia areata reported that MLR might represent a potential diagnostic marker [10]. In the present study, MLR values differed significantly between the AV patients and the control group. However, no association was found between MLR and the severity of AV. Nevertheless, we think that further, more extensive studies are now needed to evaluate the exact relationship between MLR and the severity of the disease. Studies examining the NLR in AV also appear in the literature. One such study determined no significant relationship was found between NLR and severity of AV [11]. In contrast, another study reported higher NLR values in patients with AV than in a control group [12]. No statistically significant relationship between NLR and AV severity was observed in the current research. However, considering that NLR has previously been proposed as an inflammatory marker in some inflammatory skin diseases, such as lichen planus [13] and psoriasis [14], we think that further investigation of NLR is warranted in terms of predicting the severity of AV. Another parameter evaluated in this study was the LAR. We encountered no studies specifically focusing on this parameter in AV, nor examining it in any other disease. Only one study has investigated a combination of lymphocyte and albumin level values as a prognostic biomarker in patients with rectal carcinoma. Those authors concluded that the LAR is capable of use as a prognostic biomarker in stage II-III rectal carcinomas [15]. LAR values in the current research differed significantly between the AV and control groups. However, no association was found between LAR and acne severity. Nevertheless, considering the difference between our AV and control groups, we suggest that further studies might usefully explore its potential as a novel inflammatory biomarker in AV. Another parameter that has not to date appeared in the literature is MCR. The CRP/lymphocyte ratio was examined in patients with COVID-19 patients in one study and was described as a good marker in terms of reflecting pneumonia [16]. Another study showed that the lymphocyte/CRP ratio might represent a new prognostic marker in patients with colorectal carcinoma [17]. In contrast to those studies, we investigated MCR, which differed significantly between the patients with AV and the control group. Additionally, we observed a close relationship between MCR and the severity of AV. Once further studies have been conducted, we conclude that MCR may constitute a novel biomarker capable of use in predicting the severity of AV.

Strengths and limitations

An important limitation of this study is its single-center design and relatively small number of participants, which may affect the generalizability of the results and may not be representative of the broader population. Therefore, multicenter studies with larger participation are needed to confirm and expand these findings. Another limitation is that the etiological underlying pathology of our results is not known and was not investigated in this study. However, investigating inflammatory parameters such as CAR, LAR, and MCR for the first time in patients with AV and examining their possible relationship with the disease severity suggests that our study will make a contribution to the current literature. Methodologically, taking the history of antibiotic and anti-inflammatory drug use as an exclusion criterion contributes to the reliability of the data obtained.

## Conclusions

In this study, CAR and various hematological parameters were compared in the healthy group and AV patients, and the utility of these parameters in determining the severity of acne was investigated. Significant differences emerged between the two groups’ CRP, CAR, MLR, LAR, and MCR values. The cut-off value for the CAR ratio was found to be 0.236, with a sensitivity of 68.6% and a specificity of 68.9%. Regarding the severity of acne, CAR and MCR were shown to be associated, and indicated that they may constitute inflammatory markers reflecting the severity of inflammation in AV.
